# First Spanish study on the effectiveness of ultrasound-guided sacroiliac joint injection in patients with spondyloarthritis

**DOI:** 10.1093/rap/rkac036

**Published:** 2022-05-23

**Authors:** Marco A Ramírez Huaranga, David Castro Corredor, Angel E Plasencia Ezaine, Marco Paulino Huertas, Rocío Arenal Lopez, Joaquín Anino Fernández, Claudia C Ramos Rodríguez

**Affiliations:** 1 Interventional Rheumatology; 2 Rheumatology Department; 3 Pathology Department, Hospital General Universitario de Ciudad Real, Ciudad Real, Spain

**Keywords:** spondyloarthritis, sacroiliitis, ultrasound-guided injection, disease activity

## Abstract

**Objective:**

The aim was to assess clinical improvement after US-guided injection of CSs into the SI joint of patients with SpA.

**Methods:**

This was an observational, descriptive, retrospective study of patients with SpA and sacroiliitis who received an US-guided injection into the SI joint between 1 June 2020 and 31 May 2021. Means were compared using Student’s paired *t*-test for the variables visual analog scale (VAS), BASDAI, ASDAS, CRP and ESR before and after the procedure. We evaluated the association between these variables and the clinical response using the odds ratio.

**Results:**

We analysed 32 patients with SpA [age 42.69 (8.19) years; female sex, 56.25%], with a VAS score of 7.88 (0.79), BASDAI of 5.43 (1.48) and ASDAS of 3.27 (0.86) before the procedure. At 2–3 months, 75% of patients had improved: VAS 3.81 (2.33) (−4.07, *P* < 0.0001) and BASDAI 3.24 (1.6) (−2.19, *P* < 0.0001). At 5–6 months, 59.37% had improved: VAS 4.63 (2.31) (−3.25, *P* < 0.0001), BASDAI 3.57 (1.67) (−1.86, *P* < 0.0001) and ASDAS 2.27 (0.71) (−1.0, *P* < 0.0001). Bone marrow oedema resolved in 87.5% of cases compared with the previous MRI scan. No significant association was identified with the clinical response to the injection.

**Conclusion:**

US-guided injection of CSs into the SI joint of patients with SpA and active sacroiliitis leads to an improvement in symptoms that is maintained at 5–6 months. The procedure is effective, safe, inexpensive and easy to apply.

Key messages

- This is the first study carried out in Spain on this procedure in this group of patients and whose outcome variables include activity measurements.
- US-guided CS infiltration of the SI joint in patients with SpA could be a therapeutic alternative for those in whom clinical and radiological sacroiliitis predominates.
- The incorporation of US into daily clinical practice would allow the performance of this procedure.

## Introduction

The term SpA covers a group of chronic inflammatory rheumatic diseases that mainly affect the axial skeleton [1]. The worldwide prevalence of SpA is estimated to range between 0.23 and 1.8% [2]. The disease most typically affects the SI joints, where it first manifests in 75% of patients [3]. Sacroiliitis can be the main manifestation or a complication of any of the variants of SpA.

Imaging-guided CS injection has been considered an effective therapy for >20 years [4]. This approach provides a significant improvement in pain and inflammation for ≤1 year in patients who do not have SpA [5, 6].

The few studies performed in patients with SpA have mainly evaluated the improvement in pain using a visual analog scale (VAS) and are limited by the need for an operating room and guidance based on fluoroscopy, tomography or magnetic resonance [7–9]. The advent of new US devices has meant that during the last decade, alternative approaches have been proposed for US-guided injection of the SI joint. These are all highly effective, accurate and safe. The technique is relatively simple to perform and accurate, with no need for an operating room or exposure to ionizing radiation [10–13]. Therefore, US-guided injection is now considered a safe, valid and easy technique.

The primary objective of this study was to determine clinical improvement (BASDAI, ASDAS, acute-phase reactants and the VAS score) after US-guided injection of CSs into the SI joint of patients with SpA.

## Methods

We performed an observational, descriptive and retrospective study at Ciudad Real General Teaching Hospital (HGUCR), Ciudad Real, Spain.

We reviewed the register of procedures carried out between 1 June 2020 and 31 May 2021 and included all patients fulfilling the Assessment of SpA International Society (ASAS) classification criteria for SpA with inflammatory low back pain resulting from sacroiliitis despite treatment with NSAIDs, biologics, or both. We excluded patients whose clinical history did not contain the study variables and in whom modifications to pharmacological treatment could have reduced the effectiveness of the injection.

**Fig. 1:**
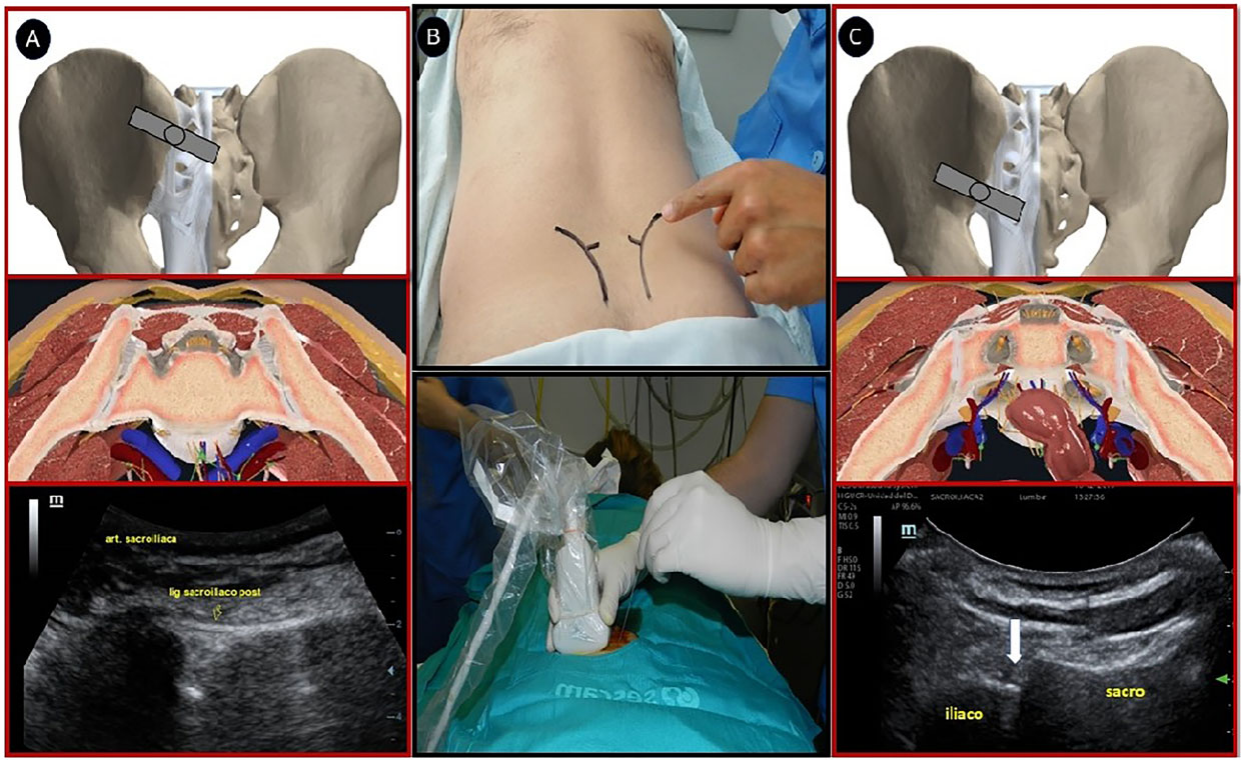
Technique used for US-guided injection into the SI joint (**A**) Anatomical and US image when approaching the upper two-thirds of the SI joint (syndesmosis). (**B**) Positioning of the patient and approach in the joint plane with a curved transducer. (**C**) Anatomical and US image when approaching the lower one-third of the SI joint (synovial).

All patients underwent US-guided injection of the SI joint with 12 mg betamethasone chronodose (6 mg acetate/6 mg phosphate) in each joint. The patient was placed in the prone position, with a pillow underneath the abdomen to minimize lumbar lordosis. A low-frequency curvilinear transducer was used. The transducer was placed transversely over the lower part of the sacrum (at the level of the sacral hiatus), and the lateral edge of the sacrum was identified. The transducer was moved laterally and cephalad until the bony contour of the ileum was clearly identified. The cleft seen between the medial border of the ileum and the lateral sacral edge represented the SI joint. A 22-gauge needle was inserted at the medial end of the transducer and advanced laterally under direct vision in plane with the US beam until it was seen entering the joint (Fig. 1).

We recorded data for the study variables (age, sex, type of SpA, time since diagnosis, HLA B27, SI joint X-rays, sacroiliac MRI scans, pharmacological treatment, VAS score, BASDAI, ASDAS, CRP and ESR) from the clinical history before the procedure and for 6–8 months after it. The outcome measures were the presence of adverse effects/complications, reduction in the VAS score for inflammatory pain (>0 = 3 points), reduction of ≥1.1 points in the ASDAS, reduction of ≥2 points in the BASDAI, and reduction of CRP and ESR values. These data were analysed by comparing means (Student’s *t*-test) for the variables VAS, BASDAI, ASDAS, CRP and ESR. The potential association between the variables and a favourable clinical response was evaluated by calculating the odds ratio. All analyses were performed with a 95% CI using StatPlus v.7.3.32 from AnalystSoft Inc. (USA).

The protocol was approved by the Research and Ethics Committee of Integrated Care Management, Ciudad Real, Spain.

## Results

Of a total of 47 patients, we included 32 for the final analysis. Table 1 shows the clinical and serological characteristics, radiographic findings, treatments and procedures.

**Table 1:** Clinical profile of patients

Variable	*n* (%)
Sex	
Male	14 (43.75)
Female	18 (56.25)
Age, mean (s.d.), years	42.69 (8.19)
Type of SpA	
Ankylosing	14 (43.75)
Non-radiographic	13 (40.62)
IBD associated	3 (9.38)
Psoriatic	2 (6.25)
Time since diagnosis, mean (s.d.), years	3.94 (2.55)
Presence of HLA B27 (+)	14 (43.75)
X-ray of SI joint according to New York criteria	
Grade 0	8 (25)
Grade 1	7 (21.88)
Grade 2	13 (40.63)
Grade 3	4 (12.49)
Grade 4	0 0
Sacroiliitis on the MRI according to ASAS criteria	
Right	7 (21.87)
Left	7 (21.87)
Bilateral	14 (43.75)
Negative	4 (12.51)
Extra-axial manifestations	16 (50)
Peripheral involvement	10 (31.25)
IBD	3 (9.38)
Psoriasis	2 (6.25)
Uveitis	1 (3.13)
Treatment	
Non-opiate analgesics	10 (31.25)
Opiate analgesics	15 (45.87)
NSAIDs	17 (53.12)
CSs	8 (25)
DMARDs	10 (31.25)
MTX	5 (15.62)
SSZ	3 (9.38)
Mesalazine	1 (3.13)
AZA	1 (3.13)
Biologics	18 (56.25)
Adalimumab	8 (25)
Golimumab	5 (15.62)
Etanercept	2 (6.25)
Secukinumab	2 (6.25)
Vedolizumab	1 (3.13)
Previous ASDAS, mean (s.d.)	3.27 (0.86)
Grade of disease activity	
Inactive (<1.3)	0 (0)
Low–moderate (<2.1)	2 (6.25)
High (≤3.5)	19 (59.37)
Very high (>3.5)	11 (34.38)
Previous BASDAI, mean (s.d.)	5.43 (1.48)
Degree of activity	
Low (BASDAI <4)	2 (6.25)
High (BASDAI ≥4)	30 (93.75)
Previous CRP, mean (s.d.) (normal, ≤0.5 mg/dl)	0.61 (0.78)
Previous ESR, mean (s.d.) (normal, ≤10 mm)	10.88 (8.94)
VAS score for previous low back pain, mean (s.d.)	7.88 (0.79)
Injection into SI joint	
Right	10 (31.25)
Left	8 (25)
Bilateral	14 (43.75)

ASAS: Assessment of SpondyloArthritis International Society; VAS: visual analog scale.

We observed an improvement in 75% of patients at 2–3 months, with a mean (s.d.) reduction of ≥3 points in the VAS for low back pain [3.81 (2.33)] (−4.07, *P* < 0.0001) and an improvement in the BASDAI value in 65.62% [3.24 (1.6)] (−2.19, *P* < 0.0001). At ∼6 months, the improvement in pain remained unchanged, at 59.37% for the VAS [4.63 (2.31)] (−3.25, *P* < 0.0001), 46.87% for BASDAI [3.57 (1.67)] (−1.86, *P* < 0.0001) and 43.75% for ASDAS-CRP [2.27 (0.71)] (−1.0, *P* < 0.0001), whereas only 18.75% experienced a considerable improvement in ASDAS-CRP (reduction ≥2). Most patients reported some degree of subjective benefit of the procedure for ≤8 months. No very significant reductions were recorded for acute-phase reactants: CRP, 0.28 (0.33) (−0.33, *P* = 0.0312); and ESR, 7.91 (6.55) (−2.97, *P* = 0.1346). After the procedure, only eight patients had undergone a follow-up MRI scan of the SI joint at 5.88 (1.25) months. Compared with the previous scan, bone marrow oedema had resolved in 87.5%.

Analysis of factors potentially associated with improved clinical response, as measured using the VAS, BASDAI and ASDAS, did not reveal a significant association with the variables analysed [age, time since diagnosis, grade of sacroiliitis by radiography, presence of HLA B27 (+), peripheral and extra-articular manifestations, and type of treatment]. However, all the patients whose symptoms improved (VAS score, BASDAI and ASDAS) had had bone marrow oedema on their MRI scan <1 year before the treatment.

Only five patients experienced injection site pain, which lasted 1–2 days. No other adverse effects were reported.

## Discussion

In the 1990s, Maugars *et al.* [7] first reported that fluoroscopy-guided injection of CSs into the SI joints of patients with SpA reduced pain by 50–64%. In the first double-blind study [14] (CSs *vs* placebo injection), the authors observed a significant improvement in the VAS (*P* < 0.05), with a 58–70% reduction in the group that received CSs [6.8 (0.6) to 1.3 (0.3)] until 6 months, whereas no significant reduction was observed in the placebo group [7.0 (0.6) to 5.2 (0.5)]. Subsequent studies with fluoroscopy-guided injection reported similar results, mainly with respect to the VAS score [15].

Braun *et al.* [8] reported their findings with a CT-guided procedure, namely, an 83.3% improvement in the VAS score (from 8.5 to 3 points), which remained at 8.9 (5.3) months, and an improvement in inflammation assessed using MRI in ∼50% of patients. Other studies with CT guidance reported similar results, with significant improvement in symptoms (*P* < 0.05) based on the VAS score and in inflammation based on MRI at 8 months [16, 17]. Various studies using MRI reported an improvement in the VAS score over a mean of 11–12 months in 78–90% of patients with SpA [9], and bone marrow oedema resolved completely in 38% and partially in 89–100% of patients [18, 19]. Studies on US-guided injection of CSs into the SI joint of patients with SpA reported a 80–100% improvement in inflammatory low back pain (VAS, 6.9 to 3.9) at 3 months (*P* < 0.05) [13, 20].

A systematic review of PubMed performed in 2019 to identify articles on injection of CSs into the SI joint of patients with SpA/sacroiliitis dating from 1990 yielded 46 references, corresponding to 468 injections in 268 patients. Inflammatory pain and stiffness improved in 80% of patients, with a mean duration of 6–8 months. The factors associated with an improved response were shorter time since diagnosis and MRI-based evidence of bone marrow oedema in the joint at baseline [21]. Finally, a recent study published in 2021 [22] showed an improvement in low back pain according to the VAS score, which was 90% at 1 month and was maintained in 68.4% at 6 months after fluoroscopy-guided injection into the SI joint in patients with SpA. The difference was significant with respect to patients who continued with their habitual treatment with NSAIDs, anti-TNF-α agents, or both, although no differences were detected in either group with respect to the improvement in BASDAI, CRP and ESR.

Our results are consistent with those reported elsewhere, indicating reduced low back pain (VAS score) in 75% and reduced disease activity, which is clearly significant during the first 2–3 months and is maintained at ∼6 months according to the VAS score (−3.25, *P* < 0.0001), BASDAI (−1.86, *P* < 0.0001) and ASDAS (−1.0, *P* < 0.0001). In line with other authors, we recorded no significant changes in levels of CRP or ESR. However, bone marrow oedema resolved in 87.5% of patients who underwent MRI after the procedure (Supplementary Table S1, available at *Rheumatology Advances in Practice* online).

Our results enable us to conclude that US-guided injection of the SI joint is a safe, effective, inexpensive and easy complementary technique in patients with SpA and recent-onset sacroiliitis that does not respond to standard treatment. Therefore, procedures of this type should be added to the rheumatologist’s therapeutic arsenal.

## Supplementary Material

rkac036_Supplementary_Data
