# Supplementary material for: First Spanish study on the effectiveness of ultrasound-guided sacroiliac joint injection in patients with spondyloarthritis
Source: Rheumatol Adv Pract. 2022 May 23;6(2):rkac036. doi: 10.1093/rap/rkac036 (PMC9154057; doi:10.1093/rap/rkac036)
Supplement: rkac036_Supplementary_Data [file rkac036_supplementary_data.doc]

| Study | Maugars et al. | Braun et al. | Pereira et al. | Fisher el at. | Fritz et al. | Sadreddini et al. | Hartung et al. | Klauser et al. | Kokar et al. | **Ramirez et al.** |
| --- | --- | --- | --- | --- | --- | --- | --- | --- | --- | --- |
| Year | 1996 | 1996 | 2000 | 2003 | 2005 | 2009 | 2010 | 2016 | 2021 | 2021 |
| No. of patients | 6 | 30 | 10 | 89 | 26 | 29 | 14 | 13 | 22 | 32 |
| Control group | 4 (placebo) | No | No | Yes (only NSAIDs) | No | No | No | No | 21 (NSAIDs/anti-TNF-α) | No |
| Technique | Fluoroscopy | Tomography | Magnetic resonance | Tomography | Magnetic resonance | Anatomic references | Ultrasound | Ultrasound | Fluoroscopy | Ultrasound |
| Improvement | 70% | 83.3% | 100% | 87.5% | 85% | 100% | 100% | 100% | 68.4 - 90.9% | 59.37-75% |
| Study variables | VAS: 6.8±0.6 to 1.3±0.3 | VAS: 8.5 a 3 | VAS: 7.7±0.6 to 3.1±1.7 | VAS: 6.9±3.4 to 1.8±1.7 | VAS: 8 to 3. | VAS: 7 to 2.3  Stiffness: 43 to 36 min. | VAS: 6.9 to 3.9 | VAS: 9.44±1.097 to  0.56±1.097 | Reduction >50% in VAS:  Injection group :68.4-75%  Noninjection group: 44.4-52.4%  No difference in BASDAI, CRP, ESR | **VAS:**  7.88±0.79 to 3.81±2.33 (3 mo) and 4.63±2.31 (6 mo)  **BASDAI:**  5.43±1.48 to 3.24±1.6 (3 mo) and 3.57±1.67 (6 mo)  **ASDAS:**  3.27±0.86 to 2.27±0.71 (6 mo)  No difference in CRP, ESR |
| Resolution of edema (MRI) | Not performed | 50% | 60% partial or total | No | 62% partial or total | 56.25% | No | No. | No. | 87.5%  (7/8 patients) |
| Duration of effectiveness | 3 mo (62%)  6 mo (58%) | 8.9±5.3 mo | 3.5±5.4 mo | 12±6 mo | ±12 mo | Follow-up 20 wk | Follow-up 1 mo | 3 mo | 12±6 mo | 6-8 mo |
| Complications | No | No | No | No | No | 8: headache, palpitations, vertigo. | No | No | No | 5: pain at injection site |

**Supplementary Table S1. Summary of the main studies on injection of corticosteroids into the sacroiliac joint in patients with spondyloarthritis.**
